# Perceived parental rejection and mental health in Italian early adolescents: the mediating role of alexithymia

**DOI:** 10.3389/fpsyg.2026.1816849

**Published:** 2026-09-23

**Authors:** Matteo Angelo Fabris, Elżbieta Zdankiewicz-Ścigała, Claudio Longobardi, Dawid Konrad Ścigała

**Affiliations:** 1 Department of Psychology, University of Turin, Turin, Italy; 2 Faculty of Psychology, SWPS University, Warsaw, Poland; 3 Department of Psychology, University of Turin, Turin, Italy; 4 Institute of Psychology, The Maria Grzegorzewska University in Warsaw, Warsaw, Poland

**Keywords:** alexithymia, externalizing symptoms, internalizing symptoms, maternal rejection, paternal rejection, perceived parental rejection

## Abstract

**Introduction:**

Grounded in Interpersonal Acceptance–Rejection Theory (IPARTheory), this study examines the associations between perceived parental rejection, alexithymia, and mental health outcomes during early adolescence. Perceived rejection from caregivers has been consistently linked to emotional and behavioral maladjustment. Alexithymia, characterized by difficulties in identifying and describing emotions, may represent a key psychological process through which parental rejection affects adolescents’ mental health. The present study aims to investigate whether alexithymia mediates the relationship between perceived maternal and paternal rejection and internalizing and externalizing symptoms.

**Methods:**

Participants were Italian early adolescents aged 10–14 years (both males and females) recruited from middle schools in Northern Italy. Adolescents completed self-report questionnaires assessing perceived parental rejection, alexithymia, and psychological symptoms. Perceived maternal and paternal rejection were measured using the Parental Acceptance–Rejection Questionnaire (PARQ). Alexithymia was assessed with the Toronto Alexithymia Scale–20 (TAS-20), while internalizing and externalizing symptoms were evaluated using the Strengths and Difficulties Questionnaire (SDQ). Mediation analyses were conducted to test the indirect effects of parental rejection on psychological symptoms through alexithymia.

**Results:**

Results showed that higher levels of both maternal and paternal rejection were significantly associated with increased internalizing and externalizing symptoms. Moreover, perceived parental rejection was positively associated with alexithymia. Mediation analyses indicated that alexithymia significantly mediated the relationships between parental rejection and both internalizing and externalizing symptoms.

**Conclusion:**

These findings highlight alexithymia as a relevant psychological mechanism linking perceived parental rejection to adolescent mental health problems, underscoring the importance of family-focused and emotion-focused preventive interventions during early adolescence.

## Introduction

Early adolescence is a period of development considered critical for the onset of psychological distress, due to the sudden physical, cognitive, emotional, and social changes that pose a major challenge to individuals’ psychological adaptation (Rapee et al., 2019). Although adolescents become increasingly independent and their peer group becomes their main social reference point, the parental relationship continues to make an important contribution to their mental well-being during this period (Badenes-Ribera et al., 2019; Hu et al., 2024). In particular, when adolescents experience a secure parental relationship characterized by affection, closeness, support, and encouragement of their autonomy, they tend to report greater emotional well-being, while they report increased psychological distress when they perceive negative relationships characterized by criticism, rejection, and disregard for their emotional needs (Badenes-Ribera et al., 2021; Fabris et al., 2025). Based on IPARTheory (Rohner, 2021), this study focuses on the perception of parental rejection and its association with adolescents’ psychological well-being, exploring the possible role of alexithymia as a potential mediating factor in this relationship.

### Perceived parental rejection and mental health

It is widely hypothesized in the literature that parenting practices influence the development and psychological well-being of children (Jugovac et al., 2022). One factor that negatively influences the perception of the quality of parental relationships and promotes insecure attachment to the caregiver is parental rejection (Ildiz and Ayhan, 2020). According to the Interpersonal Acceptance-Rejection Theory (IPARTheory; Ali et al., 2015), “parental warmth” is a key factor in promoting positive psychological adjustment in both children and adults. According to this theoretical perspective, “parental acceptance” lies at one end of the warmth dimension, while “parental rejection” lies at the other end (Rohner et al., 2005). Parental acceptance encompasses the warmth, affection, love and interest that parents show their children and is expressed by parents through behaviors such as hugging, kissing, stroking, comforting and positive words. Parental rejection describes the situation in which parents do not accept or do not want their children and perceive them as a burden (Rohner et al., 2005), which manifests itself in behaviors such as coldness, withdrawal of love, neglect, physical injury, humiliation and criticism.

Current research suggests that in various cultures, perceived parental rejection is a risk factor for the onset of psychological symptoms in children and adolescents (Fabris et al., 2026; Khaleque and Rohner, 2002). Adolescents are the active recipients of parental behavior, and the way adolescents interpret this behavior will affect their outcomes. If adolescents feel that their emotional needs for love, care, respect and comfort are met in their relationship with their parents, they are more likely to feel accepted, which may lead to better psychological adjustment. In contrast, if their emotional needs are not met, they are more likely to feel emotionally deprived and worthless. In this sense, adolescents who have experienced rejection in the parental relationship tend to develop a more negative view of themselves and the social world and report a higher risk of developing psychological symptoms (Mendo-Lázaro et al., 2019; Khaleque and Rohner, 2002). In particular, both longitudinal (Rothenberg et al., 2022) and cross-sectional studies (Fabris et al., 2025; Naz, 2012; Knappe et al., 2022) conducted in different cultural contexts report that both perceived maternal and paternal rejection predict increased internalizing symptoms in adolescence. In a relational context with parents who are perceived as rejecting, it is possible that adolescents feel unsupported and unloved and internalize negative self-images and beliefs related to the risk of being rejected and abandoned by others, which increases the development of internalizing symptoms (Badenes-Ribera et al., 2021; Knappe et al., 2022). This is a particularly critical aspect, especially in early adolescence, where belonging to a peer group becomes an important element and predicts better psychological adjustment, reducing feelings of loneliness and promoting positive emotions (Fabris et al., 2024; Longobardi et al., 2020). In addition to internalizing symptoms, the literature shows that perceived parental rejection is strongly associated with an increase in externalizing symptoms (Rothenberg et al., 2022), including ADHD symptoms (Brinksma et al., 2023), aggressive behaviors, and conduct disorders (Di Giunta et al., 2022; Folker et al., 2024). This effect is observed for both mothers and fathers, and even one rejecting parent increases risk (Miranda et al., 2016). Furthermore, some longitudinal data suggest that not only a high level of perceived parental rejection, but also fluctuations in parental acceptance and rejection appear to promote the occurrence of externalizing disorders (Folker et al., 2024). It is possible that adolescents in a negative relationship with their parents internalize aggressive behavior patterns. When adolescents perceive a rejecting parental relationship, they may also develop negative feelings and high levels of frustration, which may manifest in aggressive and socially unacceptable behavior (Hale et al., 2005; Hale et al., 2008). It is possible that a rejecting parental relationship tends to create a relational context characterized by emotional insecurity, preventing children from developing emotion regulation strategies and social skills that can reduce the risk of developing externalizing behaviors (Rothenberg et al., 2022).

Although the literature suggests that the perception of parental rejection is associated with a general deterioration in psychological well-being in adolescents, with an increase in internalizing and externalizing symptoms, much remains to be explored regarding the mechanisms underlying this relationship. In particular, it is important to identify the mediating factors that may explain how perceived parental rejection affects adolescent mental health and thus contribute to the development of more targeted and effective interventions.

Among the various aspects involved, the role of difficulties in emotional functioning, particularly in identifying and regulating emotions, stands out as a transdiagnostic factor associated with both internalizing and externalizing symptoms (Compas et al., 2017; Pandey et al., 2011). Adaptive emotional functioning is recognized as a fundamental pillar of psychological well-being (Mónaco et al., 2019). The literature also emphasizes how the quality of the relationship with caregivers influences the development of better emotion regulation skills and, consequently, more adaptive emotional functioning (Gautam et al., 2025). With this in mind, our study will seek to investigate the possible mediating role of alexithymia in the relationship between parental rejection and mental health in early adolescents.

### Alexithymia and mental health

The term alexithymia, first introduced into the literature by Sifneos (1973), refers to a cognitive-affective disorder characterized by a diminished ability to identify and describe feelings, diminished imagination, and a concrete and externally-oriented way of thinking (Taylor, 1984; Taylor et al., 1991). Alexithymia is currently conceptualized as a condition characterized by a deficit in the sufferer’s awareness and verbal reporting of feelings, reflecting a disorder of emotion regulation (Paniccia et al., 2018; Scigala et al., 2024). It is estimated that between 7 and 21% of adolescents in the community exhibit high levels of alexithymic symptoms, a percentage that rises to 85% in institutionalized adolescents with previous traumatic experiences (Muzi, 2020). Younger adolescents appear to be more at risk, while mixed results have been found in relation to gender (Prino et al., 2019). Alexithymia is considered a risk factor for the onset of psychopathological symptoms in adolescence, including internalizing and externalizing disorders (Hamel et al., 2024; Mannarini et al., 2018; Muzi, 2020; Prino et al., 2019). In general, clinicians and researchers suggest that individuals with high levels of alexithymia tend to have a reduced ability to cognitively process their emotions (Hamel et al., 2024; Taylor et al., 1991). These difficulties would lead to alexithymic individuals being particularly vulnerable to negative emotions and resorting to dysfunctional behaviors (represented by internalizing and externalizing symptoms) as a strategy for coping with emotional distress. In this context, evidence suggests that alexithymia mediates the relationship between past traumatic or dysfunctional experiences with attachment figures and an increase in psychological symptoms, both internalizing and externalizing (Hamel et al., 2024; Sharma et al., 2024). It is therefore possible that negative experiences reduce an individual’s ability to recognize and regulate their emotions, increasing the risk of developing alexithymic traits, which in turn become a risk factor for the development of psychological problems. In this regard, parental rejection is considered a form of emotional abuse that has significant implications for the psychological well-being of adolescents (Fabris et al., 2025). However, little research has been conducted on the possible mediating role of alexithymia between perceived parental rejection and the development of internalizing and externalizing symptoms in adolescents.

### Alexithymia as a potential mediating factor

There is a broad consensus in the literature that the quality of the relationship with parents in early childhood plays a role in the development of an individual’s emotional functioning (Cooke et al., 2019; Scigala et al., 2021; Scigala et al., 2022). In summary, when a parent is emotionally available, the child feels supported in regulating their emotions by recognizing their feelings, modeling emotion regulation strategies and teaching these strategies to the child (Vandewalle et al., 2017). In contrast, if a parent is emotionally unavailable or rejecting, the opportunities to learn appropriate emotional knowledge from the parent are reduced, putting the individual at high risk of mismatching somatic sensations, emotional symbols and linguistic representations and thus the risk of developing alexithymic symptoms. It is even possible that a child who is in an unsupportive or rejecting relationship with their caregiver will resort to emotional suppression as a strategy to reduce their emotional distress (Di Giunta et al., 2022). In this context, a longitudinal study conducted by van Strien et al. (2019) showed that adolescents who had a negative relationship with their parents tended to report emotional suppression more frequently, which in turn was associated with an increase in alexithymic symptoms. It is therefore possible that adolescents with a perceived negative relationship with their parents have learned to suppress and internalize their distressing emotions rather than express and communicate them, with inwardly directed feelings and somatic complaints thus fueling the development of alexithymic syndrome (Huang et al., 2022). We must bear in mind that parental rejection is comparable to a form of emotional abuse in which the child’s need for support and emotional closeness is largely ignored, and it is possible that in such a relational context the adolescent has learnt to suppress their emotions, which increases the risk of developing alexithymia. In studies with adults, particularly those with substance dependence, a significant association has been found between perceived parental rejection and alexithymia (Huang et al., 2022; Torrado et al., 2013). In particular, perceived parental rejection appears to predict the two sub-dimensions of alexithymia, namely the difficulty in recognizing one’s own feelings and the ability to verbally describe one’s own emotions (Torrado et al., 2013).

The data therefore show that alexithymia is significantly associated with both perceived parental rejection and psychological symptoms, both internalizing and externalizing. However, little is known about the possible mediating role of alexithymia between perceived parental rejection and psychological symptoms in adolescents. Previous studies have shown that among various types of adverse childhood experiences, emotional abuse is most strongly associated with alexithymic traits (Kick et al., 2024), and several lines of evidence support a significant mediating role of alexithymia between past experiences of emotional abuse and emotional neglect and psychological symptoms, both internalizing and externalizing (Brown et al., 2016; Hamel et al., 2024). However, no study has considered alexithymic symptoms in mediating the relationship specifically between parental rejection, as perceived by adolescents in their relationship with their parents, and psychological symptoms. In this vein, Fabris et al. (2025) recently published a study in which they found that in a sample of early adolescents, alexithymia played a significant mediating role in the relationship between parental rejection and symptoms of generalized anxiety disorder (GAD). However, the authors only looked at GAD, a specific disorder belonging to the class of internalizing disorders, while to our knowledge no study has extended the investigation to a broader range of psychological symptoms, including internalizing and externalizing symptoms.

### The aim of the study

In light of the considerations outlined above, the literature seems to indicate a relationship between perceived parental rejection, alexithymia and mental health, but no study that we are aware of has attempted to integrate these constructs into a single model. In particular, there is limited evidence on the potential impact of perceived maternal and paternal rejection on psychological well-being in early adolescence, a critical developmental period in which the quality of the relationship with parents plays an important role in predicting children’s psychological well-being, potentially impacting psychological adjustment in the future. Beyond treating perceived parental rejection as a unitary construct, we chose to examine maternal and paternal rejection separately, as there is theoretical and empirical reason to expect that they may relate differently to adolescents’ psychological adjustment. Consistent with IPARTheory, both parents can be sources of acceptance or rejection, but mothers and fathers often occupy distinct roles within the family system and may differ in their patterns of caregiving, disciplinary style, and emotional involvement (Rohner et al., 2005). In many cultural contexts, including Italy, mothers tend to be more centrally involved in emotional caregiving and daily discipline, whereas fathers’ involvement is often more closely tied to behavioral control and rule-setting (Bacchini et al., 2024; Fabris et al., 2026). Prior research has accordingly reported that maternal and paternal rejection can be differentially associated with internalizing and externalizing symptoms (Chen and Volling, 2023; Folker et al., 2024; Miranda et al., 2016), suggesting that collapsing maternal and paternal rejection into a single indicator could obscure meaningful gender-specific pathways. For this reason, we examined maternal and paternal rejection as distinct constructs throughout the present study. Therefore, in this study, we hypothesize that perceived parental rejection (both maternal and paternal) will be positively and significantly associated with alexithymia and internalizing and externalizing psychological symptoms. Given the differing parental roles outlined above, we further hypothesized that the strength of these associations would differ according to parent gender, although we did not specify *a priori* direction for this difference. Furthermore, we hypothesize that high levels of alexithymia will mediate the relationship between perceived parental rejection and psychological symptoms (both internalizing and externalizing).

## Methods

### Procedure and participants

The study was conducted with a large convenience sample of Italian adolescents recruited from schools in northern Italy. School principals were contacted to present the aims and methods of the study. Once authorisation was obtained from the school principal, the parents of the students received a letter introducing the study and a consent form asking for permission for their children to voluntarily participate in completing an anonymous questionnaire. Only students who had received their parents’ permission, were native Italian speakers and had sufficient reading and writing skills to take part in the study were included in the sample. Students were free to choose whether or not to participate and no rewards were used. The study complies with the guidelines of the Helsinki Declaration and the Italian Association of Psychology (AIP) and was approved by the Ethics Committee of the University of Turin. The final sample consisted of 234 Italian early adolescents aged 10 to 14 years, of whom 54.4% were girls.

### Measures

#### Socio-demographic variables

Participants completed an anonymous questionnaire containing a section of socio-demographic data that included: age, gender, living with parents; number of siblings, grade of school level.

#### Perceived parental rejection

Perceived Parental Rejection was measured using the Italian version of the Parental Acceptance and Rejection Questionnaire-child version (PARQ; Rohner and Khaleque, 2005). The PARQ is a self-report questionnaire consisting of 24 items to be answered by the subject on a Likert scale ranging from 1 (*Never*) to 4 (*Every day*). The adolescents completed both the maternal and paternal versions of the PARQ, which assess perceived maternal and paternal rejection, respectively. High scores on this measure indicate a low perception of parental rejection. The reliability of the scale for the overall score of maternal acceptance/rejection by Cronbach’s alpha measure, α = 0.84 and by McDonald’s Omega, ω = 0.84. The reliability of the scale for the overall score of paternal acceptance/rejection by Cronbach’s alpha measure, α = 0.89 and by McDonald’s omega, ω = 0.89.

#### Mental health

Early adolescents’ mental health was assessed by administering a self-report instrument, the Italian version of the Strength and Difficulties Questionnaire (SDQ) (Marzocchi et al., 2004). Students provided a rating of their psychological symptoms by answering 25 items that refer to the positive or negative feelings and behaviors. The items were evaluated on a 3-point Likert scale (i.e., not true, partially true, and absolutely true), and assessed five dimensions of students’ emotional and behavior characteristics: Emotional problems (e.g., “I am often unhappy, depressed or tearful”), conduct problems (e.g., “I get very angry and often lose my temper”), hyperactivity/inattention (e.g., “I am constantly fidgeting or squirming”), peer relationship problems (e.g., “I have one good friend or more,” reversed), and prosocial behavior (e.g., “I am helpful if someone is hurt, upset or feeling ill”). Following recommendations by Goodman et al. (2010), for the purpose of this study, items were combined to create two scores reflecting students’ internalizing symptoms (sum of emotional symptoms and problematic relationships with peers), and externalizing symptoms (sum of conduct problems and hyperactivity/inattention symptoms). The Cronbach’s alpha for both components was acceptable (internalizing symptoms: α = 0.77; externalizing symptoms: α = 0.66).

#### Alexithymia

The Italian version of the Toronto Alexithymia Scale-20 (Craparo et al., 2015) was used to assess alexithymia. The TAS-20 is a self-report measure of alexithymic symptoms that consists of four subscales: Difficulty identifying feelings (7 items); Difficulty describing feelings (4 items); Externally oriented-thinking (4 items); and Lack of subjective significance or importance of emotions (4 items). The total alexithymia score is the sum of all items, while the score for each subscale factor is the sum of specific items. The reliability of the total score by Cronbach’s alpha measure, α = 0.79 and by McDonald’s Omega, ω = 0.77.

### Data analysis

Prior to the correlational and structural analyses, the scale scores used as observed indicators were screened for missing values, univariate distributional characteristics, multicollinearity, and multivariate normality. At the level of the scale scores entered into the SEM, no missing values were present; therefore, no imputation was applied during model estimation. Skewness ranged from −1.58 to 0.48 and excess kurtosis from −0.99 to 3.55, indicating no severe univariate departures from normality. Intercorrelations among the observed indicators did not indicate problematic multicollinearity. Mardia’s critical ratios for multivariate kurtosis were 3.74 for the maternal model and 9.65 for the paternal model, indicating that multivariate normality was not fully supported. Following this, Pearson’s linear correlation coefficient was employed to explore the associations between the primary dimensions of the constructs under study and their respective subscales.

The verification of the theoretical model was conducted using structural equation modeling (SEM) in AMOS, which allows for the simultaneous testing of direct and indirect relationships within the theoretical model (Byrne, 2016). The model was based on previously identified relationships in the literature (Baron & Kenny, 1986). The presence of partial or full mediation was assessed, where the mediator (alexithymia) explained the relationship between the independent variable (Mother/Father acceptance/rejection) and the dependent variable (Strengths and Difficulties).

Given that multivariate normality was not fully supported, the indirect effects were estimated using 5,000 nonparametric bootstrap resamples, which provided bootstrap confidence intervals for statistical inference (Preacher & Hayes, 2008).

Mediation was considered significant when the confidence interval did not include zero (Shrout & Bolger, 2002). Additionally, the fit of the partial and full mediation models was assessed using the following parameters: RMSEA ≤0.06 – good fit; CFI and TLI ≥ 0.95 – acceptable fit; χ^2^/df < 3 – acceptable fit (Hu & Bentler, 1999).

The partial- and full-mediation models were evaluated as theory-based alternative specifications. The final model specifications were retained on theoretical grounds, and modification indices were not used to introduce *post hoc* paths, correlated residuals, cross-loadings, or indicator deletions. Standardized indirect effects were calculated as the product of the standardized a and b paths.

## Results

### Descriptive statistics and correlations

The initial analyses examined linear relationships among the key variables. Beginning with the relationship between acceptance in the adolescent-parent relationship and the level of alexithymia among adolescents, it was observed that the general level of acceptance correlated similarly with alexithymia for both parents: *r* = −0.35, *p* < 0.001 for the mother and *r* = −0.31, *p* < 0.001 for the father (Table 1). Lower parental acceptance was associated with higher levels of alexithymia in adolescents.

**Table 1:** Descriptive statistics of the analyzed variables and Pearson’s correlation matrix.

Variable	M	SD	MIN	MAX	2	3	4	5	6	7	8	9	10	11	12	13	14	15	16	17	18	19
1. Age	11.86	0.93	10	14	0.55	0.082	0.049	−0.085	−0.076	−0.046	−0.022	−0.047	−0.065	−0.084	−0.002	−0.051	−0.073	0.102	0.092	0.142*	0.021	0.008
2. SDQ total	22.64	6.92	10	41		0.899**	0.835**	−0.512**	−0.516**	−0.441**	−0.460**	−0.488**	−0.396**	−0.440**	−0.481**	−0.352**	−0.440**	0.669**	0.677**	0.542**	0.15*	0.329**
3. SDQ internalizing	12.24	4.35	6	25			0.511**	−0.374**	−0.401**	−0.323**	−0.387**	−0.374**	−0.338**	−0.401**	−0.375**	−0.303**	−0.391**	0.596**	0.620**	0.492**	0.089	0.293**
4. SDQ externalizing	10.61	3.46	4	22				−0.482**	−0.465**	−0.389**	−0.372**	−0.414**	−0.288**	−0.333**	−0.358**	−0.278**	−0.288**	0.574**	0.536**	0.456**	0.179*	0.299**
5. PARQ Mother acceptance rejection	58.79	6.95	33	73					0.824**	0.732**	0.315**	0.572**	0.386**	0.477**	0.381**	0.275**	0.322**	−0.347**	−0.324**	−0.293**	−0.124	−0.154*
6. PARQ warmth Mother	19.51	4.99	3	26						0.478**	0.411**	0.369**	0.385**	0.548**	0.333**	0.348**	0.267**	−0.405**	−0.359**	−0.347**	−0.162*	−0.191**
7. PARQ hostility Mother	20.26	3.47	4	24							0.580**	0.576**	0.251**	0.275**	0.496**	0.376**	0.414**	−0.253**	−0.300**	−0.181**	−0.024	−0.092
8. PARQ indifference Mother	20.10	3.78	2	24								0.600**	0.214**	0.278**	0.471**	0.499**	0.440**	−0.283**	−0.335**	−0.207**	−0.036	−0.093
9. PARQ undeferentiated rejection Mother	14.27	2.29	2	16									0.286**	0.267**	0.417**	0.289**	0.457**	−0.225**	−0.263**	−0.177**	−0.044	−0.054
10. PARQ Father acceptance rejection	57.52	9.41	21	73										0.868**	0.700**	0.524**	0.656**	−0.308**	−0.275**	−0.241**	−0.116	−0.171*
11. PARQ warmth Father	18.54	6.07	1	30											0.475**	0.497**	0.474**	−0.374**	−0.348**	−0.318**	−0.085	−0.207**
12. PARQ hostility Father	20.30	3.89	5	24												0.670**	0.658**	−0.242**	−0.266**	−0.137*	−0.048	−0.138*
13. PARQ indifference Father	19.35	4.84	3	41													0.669**	−0.271**	−0.271**	−0.187**	−0.094	−0.133*
14. PARQ undeferentiated rejection Father	13.96	2.85	2	16														−0.203**	−0.233**	−0.137*	−0.047	−0.073
15. TAS alexithymia total	43.45	14.69	10	76															0.877**	0.778**	0.280**	0.669**
16. DIF difficulty identifying feelings	12.68	7.83	0	30																0.617**	0.014	0.461**
17. DDF difficulty describing feelings	11.12	4.6	0	20																	0.06	0.375**
18. EOT externally oriented thinking	9.17	3.94	0	18																		−0.045
19. LSS lack of subjective significance or importance of emotions	10.48	4.68	0	20																		1

M, mean; SD, standard deviation; MIN, Minimum; Max, Maximum; *p < 0.05; **p < 0.001. Column numbers refer to the corresponding numbered variables in the first column.

Among the subdimensions of alexithymia, only externally oriented thinking was not significantly correlated with parental acceptance. However, there was a significant relationship between this subdimension and maternal warmth (*r* = −0.16, *p* < 0.05). In contrast, the dimensions of difficulty identifying feelings (DIF) and difficulty describing feelings (DDF) demonstrated moderate and significant correlations with parental acceptance(Table 1). Notably, for overall alexithymia scores and the DIF and DDF subscales, the associations with maternal acceptance were consistently slightly stronger than those with paternal acceptance. An opposite pattern was observed for the dimension of low social support (LSS), which significantly correlated with paternal acceptance and its three subdimensions, but only with the overall score and one subscale of maternal acceptance (Table 1).

Focusing on the relationship between parental acceptance and adolescent difficulties, the data suggest a general trend: higher parental acceptance was associated with lower levels of both externalizing and internalizing problems among adolescents.

An interesting difference emerged in the relationship between adolescent problems and parental acceptance based on the parent’s gender. Maternal acceptance and its dimensions were more strongly related to externalizing problems, whereas paternal acceptance showed a stronger relationship with internalizing problems (Table 1). Finally, it is worth noting that alexithymia and its dimensions were significantly positively correlated with both externalizing and internalizing problems reported by adolescents (Table 1).

### Verification of the theoretical model

To test the maternal model, we examined whether alexithymia mediated the association between maternal acceptance/rejection and adolescents’ strengths and difficulties. The indirect effect was significant, B_ind_ = −0.737, SE = 0.257, 95% bootstrap CI [−1.402, −0.379], *p* < 0.001; the corresponding standardized indirect effect was β_ind_ = −0.347. The direct effect remained significant after accounting for alexithymia, B = −1.018, SE = 0.229, *p* < 0.001.

Furthermore, the fit of the full and partial mediation models was assessed using the basic parameters reported in Table 2, which confirm a better fit of the partial mediation model to the data (Table 2). Therefore, it can be concluded that alexithymia partially mediates the relationship between Mother acceptance/rejection and strengths and difficulties.

**Table 2:** Fit indices of the mediation model for the mediating role of alexithymia in the relationship between Parental rejection (Mother and Father) and strengths and difficulties.

Relationship pathway		Mediation models							Direct effect	Indirect effect	95% LLCI	95% ULCI	*p* value
		χ^2^/df	CFI	TLI	RMSEA	LO 90	HI 90	*p* close					
Mother acceptance → alexithymia → strength and difficulties	Partial mediation	1.301	0.98	0.972	0.078	0.001	0.081	0.548	−1.018	−0.737	−1.402	−0.379	*p* < 0.001
	Full mediation	1.892	0.939	0.917	0.045	0.048	0.108	0.063					
Father accaptance → alexithymia → strenth and difficulties	Partial mediation	1.302	0.982	0.975	0.045	0.001	0.080	0.548	−0.508	−0.497	−0.844	−0.288	*p* < 0.001
	Full mediation	3.571	0.843	0.792	0.133	0.108	0.159	0.001					

RMSEA, Root Mean Square Error of Approximation; CFI, Comparative Fit Index; TLI, Tucker-Lewis Index; LLCI, Lower-Level Confidence Interval; ULCI, Upper-Level Confidence Interval; *p* values reported as two-tailed. Standardized indirect effects, calculated as the product of the standardized a and b paths, were β_ind_ = −0.347 for the maternal model and β_ind_ = −0.338 for the paternal model.

Focusing on the relationships between variables, the direct relationship between mother acceptance and strengths and difficulties is very strong (c = −0.83, *p* < 0.001) (Figure 1). It was then demonstrated that there is a significant relationship between the independent variable and the mediator, alexithymia (a = −0.54, *p* < 0.001). This result indicates that greater acceptance in the mother–child relationship is a protective factor against the development of alexithymia. Finally, when examining the mediating role of alexithymia, it was shown that after introducing the mediator into the model, the relationship between alexithymia and strengths and difficulties remained strong (b = 0.65, *p* < 0.001), while the direct effect weakened (c’ = −0.48, *p* < 0.001) and remained significant (Figure 1). Therefore, high levels of maternal rejection are strongly associated with both externalizing and internalizing difficulties, as well as with alexithymia, which additionally determines the development of these difficulties among adolescents.

**Figure 1:**
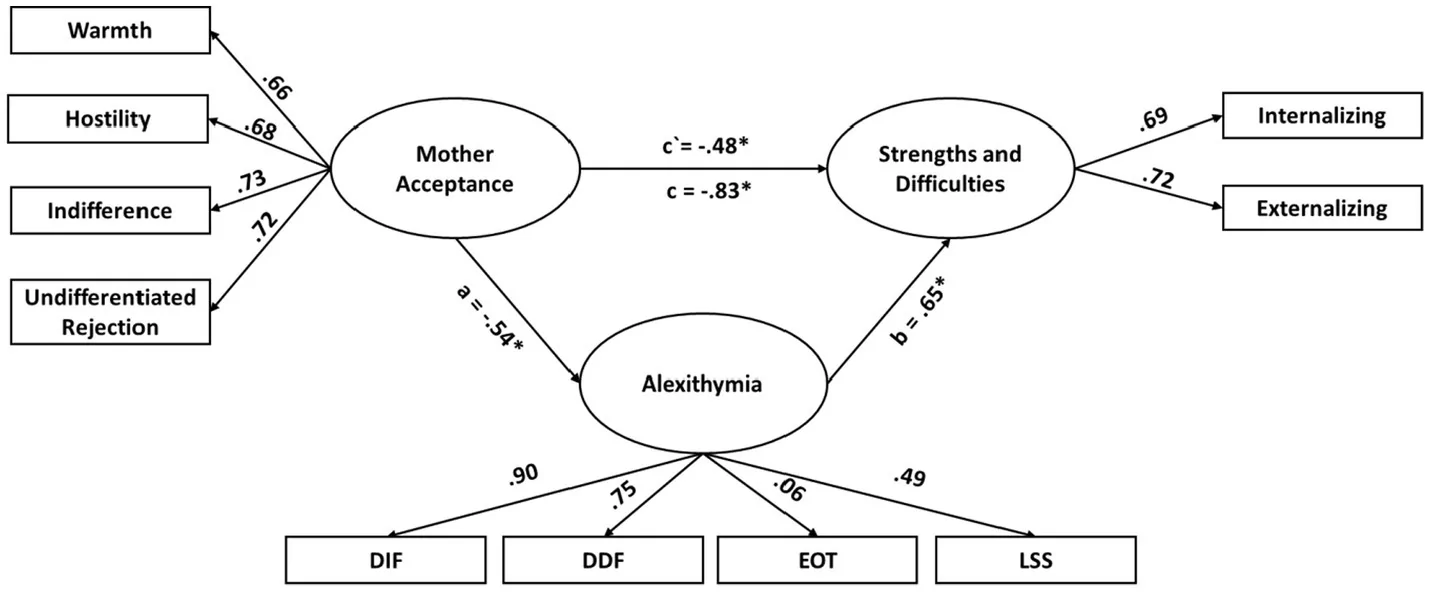
Illustration of the mediating role of alexithymia in the relationship between mother acceptance and strengths and difficulties. All values in the figure are standardized. a—Relationship between the independent variable and the mediator. b—Relationship between the mediator and the dependent variable. c—Direct relationship between the independent and dependent variables. c’—Relationship between the independent and dependent variables after accounting for the role of the mediator.

Next, we examined whether alexithymia mediated the association between paternal acceptance/rejection and adolescents’ strengths and difficulties. The indirect effect was significant, B_ind_ = −0.497, SE = 0.137, 95% bootstrap CI [−0.844, −0.288], *p* < 0.001; the corresponding standardized indirect effect was β_ind_ = −0.338. The direct effect remained significant after accounting for alexithymia, B = −0.508, SE = 0.134, *p* < 0.001.

Additionally, the fit of the full and partial mediation models to the data was assessed using the basic parameters reported in Table 2, which confirm a better fit of the partial mediation model to the data (Table 2). Therefore, it can be concluded that alexithymia partially mediates the relationship between Father acceptance/rejection and strengths and difficulties.

Focusing on the relationships between variables, the direct relationship between father acceptance and strengths and difficulties is strong (c = −0.67, *p* < 0.001) (Figure 2). It was then demonstrated that there is a significant relationship between the independent variable and the mediator, alexithymia (a = −0.46, *p* < 0.001). Finally, when examining the mediating role of alexithymia, it was shown that after introducing the mediator into the model, the relationship between alexithymia and strengths and difficulties remained very strong (b = 0.74, *p* < 0.001), while the direct effect weakened (c’ = −0.34, *p* < 0.001) and remained significant (Figure 2).

**Figure 2:**
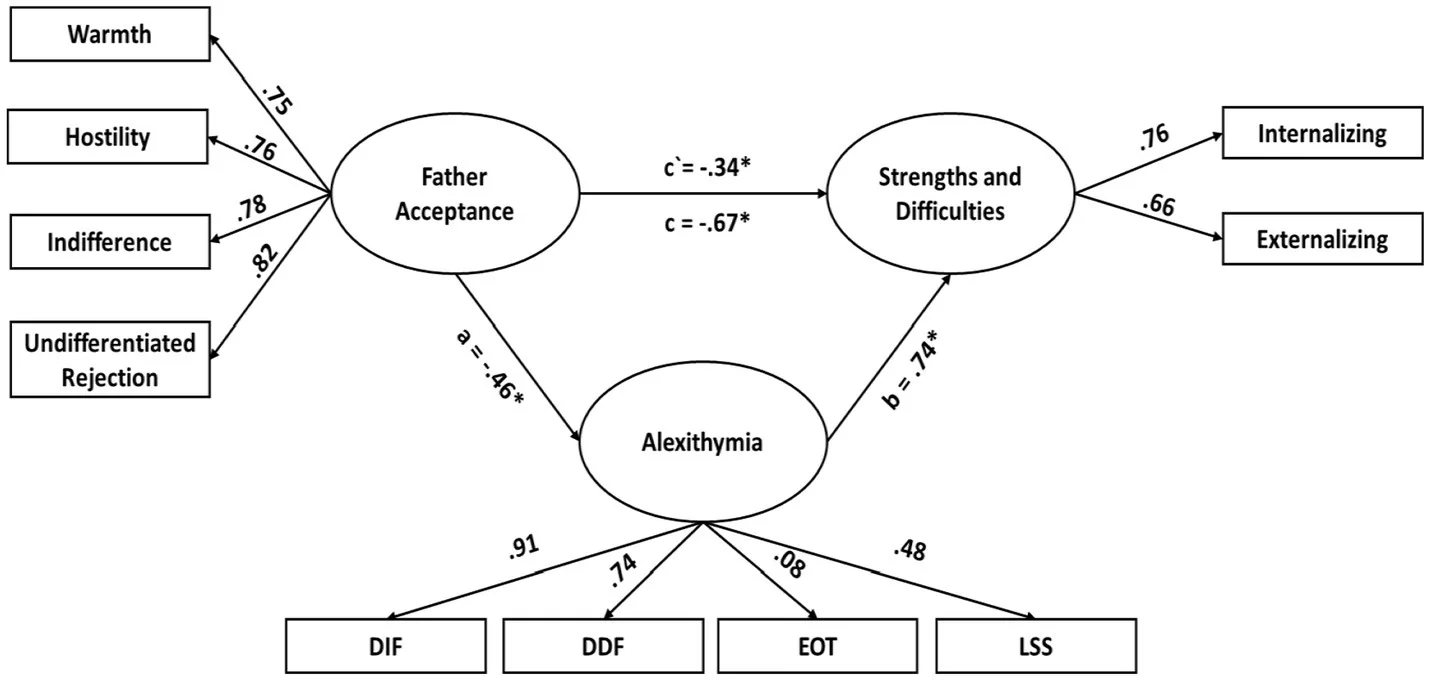
Illustration of the mediating role of alexithymia in the relationship between father acceptance and strengths and difficulties. All values in the figure are standardized. a—Relationship between the independent variable and the mediator. b—Relationship between the mediator and the dependent variable. c—Direct relationship between the independent and dependent variables. c’—Relationship between the independent and dependent variables after accounting for the role of the mediator.

In summary, when comparing both models, it can be observed that the direct relationship between rejection and strengths and difficulties is stronger in the relationship with the mother (Figure 1). By contrast, when considering the relationship between alexithymia and strengths and difficulties, a stronger association was observed in the father-child relationship (Figure 2).

## Discussion

The aim of our study was to investigate the possible role of alexithymia in mediating the relationship between perceived parental rejection (both maternal and paternal) and mental health in a sample of adolescents. To our knowledge, the relationship between each variable has been extensively examined in previous literature, but no study to date has attempted to test their relationship in a single model, particularly during early adolescence. This is a limitation, especially given the significant and potential impact of perceived parental rejection on the adolescents’ mental health.

Consistent with our hypotheses, perceived parental rejection was associated with poorer adolescents’ mental health, consistent with its hypothesized role as a risk factor, in line with recent studies from Italy reporting that both maternal and paternal rejection are associated with increased internalizing and externalizing symptoms in adolescents (Di Giunta et al., 2022; Fabris et al., 2025; Miranda et al., 2016). It is possible that an adolescent who perceives parental rejection may internalize a negative view of themselves and significant others and develop a distorted and pessimistic perception of themselves and the social world, thereby reinforcing the concerns that are central to the development of internalizing symptoms. In addition, the perception of parental rejection could increase negative feelings and frustration, which may increase the risk of impulsive and socially deviant behavior to counteract these feelings. We also observed gender-specific differences, with perceived maternal rejection more strongly associated with internalizing symptoms and perceived paternal rejection more strongly associated with externalizing symptoms. Although the literature on this point is still underdeveloped, our data appear to partially replicate the few studies available to us (Fabris et al., 2025; Folker et al., 2024). In line with the findings of Fabris et al. (2025) with Italian adolescents, our study shows a stronger association between perceived maternal rejection and internalizing symptoms than between perceived paternal rejection and internalizing symptoms. These data could be explained from a cultural point of view. It should be noted that our data do not directly test these cultural mechanisms, and the following interpretation is therefore offered as one plausible explanation rather than an empirically confirmed one. In Italy, the mother is traditionally the main person responsible for the upbringing of children, and this would allow the child to share a more intimate relationship with the maternal figure than with the paternal one, but is also more exposed to the mother’s criticism and disciplinary measures. Considering that the mother is the main source of emotional support for the child, it is conceivable that in a relationship perceived as rejecting, the adolescent may lose confidence in the attachment relationship and internalize criticism and negative thoughts about themselves that could fuel fears and anxieties, such as the fear of being imperfect, abandoned or rejected by others, thus potentially contributing to internalizing symptoms. In addition, prior research suggests that mothers may be more psychologically controlling and verbally abusive compared to fathers (Zhang and Wang, 2023), which could, in principle, fuel adolescents’ doubts about their personal qualities and lead to low self-esteem and depressive feelings, potentially increasing the risk of internalizing disorders (Zhang and Wang, 2023). We stress that this cultural explanation remains speculative in the absence of direct measures of parenting style or cultural attitudes in our own sample, and future studies should test it directly — for example, by assessing parental psychological control or disciplinary practices alongside perceived rejection.

Regarding externalizing symptoms, previous studies have highlighted that perceived paternal rejection is more strongly associated with these symptoms than perceived maternal rejection (Folker et al., 2024), whereas other studies have reported that perceived paternal rejection—but not maternal rejection—is associated with a range of externalizing symptoms (Chen and Volling, 2023; Zhang and Wang, 2023). Compared to mothers, fathers may express behavior that is perceived as rejecting by their children by more frequently using punishment strategies characterized by strict rules and increased use of corporal punishment (Abrahamyan et al., 2024; Bacchini et al., 2024). Although plausible, this explanation is drawn from the broader literature rather than from data collected in the present study and should be interpreted with caution. According to these authors, children may therefore observe and imitate such behaviors as a means of coping with interpersonal relationships and emotional stress, which could contribute to an increase in externalizing symptoms. In addition, corporal punishment and harsh discipline tend to undermine personal safety and increase emotional dysregulation, which increases the likelihood that adolescents will resort to aggressive or oppositional behaviors to cope with stress or frustration (Zhang et al., 2024). As with the maternal pathway discussed above, we did not directly measure disciplinary style or corporal punishment in our sample, and this cultural/parenting-based interpretation should therefore be regarded as a hypothesis for future research rather than a confirmed mechanism. The significant mediating role of alexithymia observed in our study is consistent with previous literature reporting that adolescents (Lukas et al., 2022) and adults (Huang et al., 2022) who perceive or recall their relationship with their parents as characterized by low levels of affection and high levels of negativity, criticism and rejection tend to report higher levels of alexithymia. This finding is consistent with developmental theories which recognize the quality of the relationship with caregivers as the foundation for positive emotional development of the individual (Spangler and Zimmermann, 1999; Scigala et al., 2021, 2022). It is possible that adolescents who grew up in a relational context in which they felt rejected by their parents are more likely to resort to emotional suppression strategies to reduce their demands for affection and the painful feelings associated with rejection and lack of support from caregivers as they develop appropriate emotional regulation strategies. In such a relational context with parents, the development of skills related to identifying and verbalizing their emotions could therefore be impaired, increasing the risk of developing alexithymic symptoms (Fabris et al., 2018, 2025; van Strien et al., 2019). Previous studies had found that alexithymia was a significant mediator in the relationship between past experiences of emotional abuse in the relationship with caregivers and psychological symptoms (Hamel et al., 2024; Kick et al., 2024), but few studies have specifically considered parental rejection. A recent Italian study (Fabris et al., 2025) reported that alexithymia statistically mediate the relationship between perceived parental rejection and internalizing symptoms. Our study extends this work by showing that alexithymia was also found to statistically mediates the relationship between perceived parental rejection and externalizing symptoms, in the same sample. In line with previous literature, alexithymia has been found to be a transdiagnostic factor in adolescents associated with both internalizing and externalizing symptoms (Hamel et al., 2024; Mannarini et al., 2018; Prino et al., 2019). It is possible that adolescents who grow up in the context of a perceived negative relationship with their parents have difficulties developing adaptive emotion regulation skills, which may increase the risk of alexithymia, which in turn could increase the risk of psychological symptoms, both internalizing and externalizing. In this sense, difficulties in identifying and verbalizing emotions may hinder alexithymic individuals from cognitively processing their emotions, which could make them more likely to adopt dysfunctional behaviors, both internalizing and externalizing symptoms, as a strategy to cope with and regulate negative and painful feelings.

### Limits and future directions

Although our study offers a new perspective in literature and makes a modest contribution to the expansion of our current knowledge in this particular area, our data must be interpreted with caution, bearing in mind the limitations of the study. Namely, our study adopted a cross-sectional approach, which prevents us from expressing ourselves in terms of linear causality. If we hypothesize that the experience of parental rejection precedes the development of alexithymia and contributes to adolescents’ psychological adjustment at the present time, we cannot rule out the possibility that negative emotional states or alexithymic traits in adolescents influence their perception of the quality of their relationship with their parents. Furthermore, there seems to be evidence that perceived parental rejection and psychological symptoms may have a bidirectional relationship and may be mutually reinforcing (Zhu et al., 2020). Future longitudinal studies could therefore strengthen these findings by contributing to the understanding of causal relationships. In addition, the perception of parental rejection, as well as the other measures considered, were exclusively self-reported by adolescents. The use of self-report instruments could lead to biases related to text comprehension, memory and social desirability. More importantly, because perceived parental rejection, alexithymia, and psychological symptoms were all assessed through the same self-report method and from the same informant (the adolescent) at a single time point, our findings may be affected by common method variance and shared source bias. This shared-method variance could have artificially inflated the associations among these constructs, including the magnitude of the indirect (mediation) effects reported here, since correlated measurement error across the independent variable, mediator, and outcome can spuriously strengthen both the a-path (parental rejection–alexithymia) and the b-path (alexithymia–psychological symptoms) simultaneously, thereby inflating the estimated indirect effect. The mediation effects observed should therefore be interpreted with this possibility in mind and may partly overestimate the true underlying relationships. Future studies may therefore seek to investigate the relationship between the constructs we examined using multi-informant or multi-method designs (for example, combining adolescent self-report with parent- or teacher-report measures of psychological symptoms, or with interview-based or observer-rated assessments of alexithymia and parenting behavior) which would help disentangle genuine associations from shared-method artifacts. A further limitation concerns the reliability of the SDQ externalizing symptoms subscale, which was relatively modest in our sample (α = 0.66) and falls below the conventional threshold of 0.70 typically considered acceptable. This comparatively low internal consistency may partly reflect the limited number of items comprising this composite score and could have introduced additional measurement error into our estimates. Therefore, the associations reported here between perceived parental rejection, alexithymia, and externalizing symptoms should be interpreted with some caution, since lower reliability tends to attenuate observed relationships and the true associations may therefore be somewhat larger than those estimated in the present study. Future research could address this issue by using expanded or alternative measures of externalizing behavior, or by examining its subdimensions (e.g., conduct problems and hyperactivity/inattention) separately, it obtain more precise and reliable estimates. Finaly, our sample is a convenience sample drawn from the general population. Future studies could replicate this investigation with representative samples of adolescents to increase the generalizability of the data in the Italian context and include clinical samples of early adolescents who have received formal diagnoses from specialists such as psychologists and psychiatrists.

### Practical implications

Our study highlights some possible practical implications. For professionals working in the field of adolescent mental health, it is important to raise parents’ awareness of the potential harmful effects of a negative parental relationship with their children. It is therefore possible to help parents understand what behaviors and signs indicate a negative relationship with their children and encourage them to seek help from mental health professionals who specialize in the developmental age. In addition, our data indicate the importance of examining the quality of the parent–child relationship at the assessment stage when an adolescent is displaying psychological symptoms, focusing particularly on perceptions of a hostile and rejecting relationship. In addition, for adolescents who have experienced a rejecting parental relationship, it is important to assess the degree of their psychological impairment and examine the possible role of alexithymia in increasing the risk of psychological symptoms in order to identify appropriate psychological intervention strategies.

## Conclusion

In conclusion, our study contributes to the current literature by extending existing knowledge on the possible correlates of perceived parental rejection with regard to psychological well-being in early adolescence. In particular, our study identified the possible mediating role of alexithymia in explaining the association between perceived parental rejection and internalizing and externalizing symptoms. Our data suggest that adolescents who perceive their relationship with their parents as rejecting have a higher risk of reporting psychological symptoms, both internalizing and externalizing. It is possible that adolescents have not received support in developing appropriate emotion regulation strategies in a relational context with attachment figures who are perceived as rejecting. Adolescents may have learned to suppress their emotions in order to distance themselves from the painful feelings associated with the relationship with their attachment figures, having difficulty recognizing and verbalizing their emotions, which contributes to the onset of alexithymic symptoms. In this way, adolescents with high levels of alexithymia who are unable to cognitively process their emotional experiences may increasingly resort to dysfunctional behaviors to cope with their emotional distress, increasing the risk of internalizing and externalizing symptoms. These symptoms may therefore represent an extreme attempt by alexithymic adolescents to cope with emotional distress and painful feelings.

## Data Availability

The raw data supporting the conclusions of this article will be made available by the authors, without undue reservation.
